# Novel phenotypic resistance to artemisinin results in reduced exposure to drug at the most sensitive stage of intraerythrocytic development in *Plasmodium falciparum*

**DOI:** 10.1186/1475-2875-13-S1-P43

**Published:** 2014-09-22

**Authors:** Amanda Hott, Debora Casandra, Kansas Sparks, Geocel Castarnes, Amanda Rutter, Dennis Kyle

**Affiliations:** 1 University of South Florida, Tampa, FL, USA

## Background

Recent emergence of artemisinin resistant *Plasmodium falciparum* in Southeast Asia has threatened malaria control efforts across the globe. Clinical resistance is characterized by reduced rates of parasite clearance following treatment; yet *in vitro* phenotypes of artemisinin resistance suitable for elucidating molecular mechanisms of resistance remain elusive.

## Materials and methods

In this study we culture adapted and characterized a series of *P. falciparum* clones from Cambodia; these included parasites from cases with reduced parasite clearance rates *in vivo*. *In vitro* artemisinin resistance phenotypes were assessed by using T_0_^3^H-hypoxanthine assays, a novel parasite clearance assay, microscopy and flow cytometry to characterize intraerythrocytic development, and limiting dilution to assess parasite viability.

## Results

Detailed phenotypic characterization of artemisinin resistant *P. falciparum* revealed a series of complex phenotypes that included 4-8 fold resistance to multiple artemisinin derivatives *in vitro* that is stable for >1 year. By using a novel parasite clearance assay we observed reduced parasite reduction rates (PRRs) for resistant clones *in vitro* and these were positively correlated with parasitemia clearance half-lives *in vivo*. Most remarkably we discovered an altered pattern of intraerythrocytic development of the artemisinin resistant clones of *P. falciparum*. In the absence of any drug pressure, resistant clones exhibited a prolonged phase of ring stage development followed by a significantly shortened trophozoite stage of development. Given that ring stages are the most resistant to inhibition by artemisinin and the trophozoites are the most susceptible, this novel phenotypic resistance to artemisinin results in reduced exposure to drug at the most sensitive stage of development (trophozoite). Additional confirmation of the selective pressure driving this phenotype is one resistant clone exhibited a 12 hour shorter erythrocytic life cycle that also compressed the trophozoite stage of development.

## Conclusions

The evolution of artemisinin resistance appears to select for altered intraerythrocytic development such that the most susceptible stage of development is significantly reduced in the cell cycle. Our data demonstrate that altered intraerythrocytic development is a novel mechanism of phenotypic resistance to artemisinin antimalarial drugs.

